# Does Therapeutic Exercise Support Improvement in Cognitive Function and Instrumental Activities of Daily Living in Patients with Mild Alzheimer’s Disease? A Randomized Controlled Trial

**DOI:** 10.3390/brainsci13071112

**Published:** 2023-07-22

**Authors:** Vasileios Papatsimpas, Sotiria Vrouva, George Papathanasiou, Marianna Papadopoulou, Christina Bouzineki, Sophia Kanellopoulou, Dimitra Moutafi, Daphne Bakalidou

**Affiliations:** 1 Physiotherapy Department, School of Health and Care Sciences, University of West Attica (UNIWA), 12243 Athens, Greece; svrouva@uniwa.gr (S.V.); gpapa@uniwa.gr (G.P.); mpapad@uniwa.gr (M.P.); dbakalid@uniwa.gr (D.B.); 2 Laboratory of Neuromuscular and Cardiovascular Study of Motion (LANECASM), School of Health and Care Sciences, University of West Attica (UNIWA), 12243 Athens, Greece; 3 Department of Physical Therapy, General Hospital of Athens G. GENNIMATAS, 11527 Athens, Greece; 4 Department of Physical Therapy, 401 Army General Hospital of Athens, 11525 Athens, Greece; 5 Alzheimer Athens Association, 11636 Athens, Greece; cbouzineki@yahoo.com (C.B.); sophia.kanellopoulou@gmail.com (S.K.); 6 Pathological Department, General Hospital Konstantopouleio, 14233 Nea Ionia, Greece; moutafdim@yahoo.gr

**Keywords:** dementia, Alzheimer’s disease, therapeutic exercise, aerobic exercise, resistance exercise, cognitive function, activities of daily living

## Abstract

This randomized controlled trial aims to investigate the effect of 12 weeks of therapeutic exercise on cognitive function and daily activities in patients with mild Alzheimer’s disease (AD). A total of 171 patients with mild AD from the Amarousion Day Care Center of the Alzheimer Society of Athens and the Athens General Hospital “G. Gennimatas” were randomly divided into three groups. Group A (aerobic and resistance exercise, *n* = 57), group B (resistance exercise, *n* = 57), and group C (control group, *n* = 57). Group A followed a weekly program consisting of 5 days with 30 min walking and 3 days with resistance exercises for about 45 min. Group B followed only a resistance exercise program, the same as group A. Group C did not participate in any exercise program. After the intervention, cognitive function was assessed with the Cognitive Examination-Revised (ACE-R), Trail Making Test A-B (TMT A-B), and Digit Span Test Forward and Backward (DST F-B) and daily activities with the instrumental activities of daily living scale (IADLs). A significant intervention effect was observed for all outcome measures (global cognitive function and instrumental activities of daily living). ANCOVA Bonferroni corrected post hoc tests revealed that the aerobic and resistance group improved compared to the control group on all measurement scales. The resistance group also showed an improvement compared to the control group. No significant effects were found between the aerobic and resistance group and the resistance group in any of the outcome measures.

## 1. Introduction

Dementia is an “umbrella” term for a series of cognitive, behavioral, and psychological symptoms of brain disease [1,2,3,4].

The main symptoms of dementia are difficulties in memory, language, problem solving, communication, personality changes, and other thinking skills that affect one’s physical function and ability to perform daily activities [1,2,3,5,6]. Activities of daily living (ADL) functions can be divided into basic ADL (BADL) (e.g., feeding, personal hygiene, and dressing) and instrumental ADL (IADL) (e.g., telephone use, shopping, and mode of transportation) [7]. IADL has been found to decline in the early stages and may be more related to cognitive abilities, whereas BADL declines in more advanced stages and may be less dependent on cognitive function [7]. Deterioration of instrumental activities of daily living occurs in the early stages of AD [8]. Furthermore, the effect of exercise on ADL in individuals with dementia has been examined only to a limited extent [9].

According to the 2021 Alzheimer’s Disease World Report, more than 55 million people worldwide are living with cognitive impairment, which is expected to reach 78 million by 2030 [10,11]. Also, the World Health Organization (WHO) reports that the number of people living with dementia is estimated to be 139 million in 2050 [12]. Dementia has many causes. Alzheimer’s disease (AD) is the most common cause of dementia and accounts for 60–80% of cases in older people (aged ≥ 65 years) [1,4,5,12,13,14,15]. AD is a progressive, irreversible neurodegenerative disease that leads to disability and causes a large socioeconomic burden [14,16,17,18] and, for this reason, it is managed in terms of an epidemic [19].

Dementia, as a leading cause of disability and dependency, has a significant impact, not only on individuals but also on caregivers, families, communities, and societies [20]. It is one of the greatest global health and social care challenges of the 21st century [20].

As such, the increasing number of persons with dementia, with its significant social and economic impact, combined with the lack of effective treatment [17,21], makes it imperative to target the reduction of modifiable risk factors for dementia.

Indeed, in recent years, nonpharmacological treatments have gained extensive attention in the treatment of AD [22], one of which is physical activity and exercise, although the relationship between physical exercise and cognitive function is controversial for some [23,24]. However, the belief that physical activity is among the modifiable risk factors in dementia is constantly gaining ground [1]. Exercise, as a low-cost and low-risk nonpharmacological intervention, has the potential to provide benefits to persons suffering from dementia; so, there is growing interest in its role [10,21,25,26,27].

The effect of exercise, as well as different programs on activities of daily living in patients with dementia, has been examined to a limited extent [9], with studies indicating that systematic exercise, through various mechanisms, promotes brain function and maintains and improves the cognitive functions [10,28,29].

Even though there have been several studies examining the effect of various types of exercise, strong recommendations cannot yet be made regarding the specific types, the frequency, or the intensity of exercise or which duration of activity could be most effective in reducing the risk of dementia, in delaying cognitive worsening or achieving possible improvement [1,4,5,21,26,30]. It is characteristic that there is a wide range of variation in the applied frequency, intensity, and duration of the exercise [10,31,32]. Aerobic exercise has been studied more where it seems to document its superiority even if there are conflicting opinions as well as mixed interventions that also seem to have positive effects [23,24,27,33,34]. Resistance exercise interventions have been less studied but have shown positive effects [27,33]. The comparison of resistance exercise with the combined intervention of aerobic and resistance exercise together has not been studied.

The aim of this study is to investigate the effect of therapeutic exercise, through different types, on cognition and activities of daily living in patients with mild AD.

## 2. Material and Methods

We performed a twelve (12) week double-blind randomized clinical trial.

**Figure 1:**
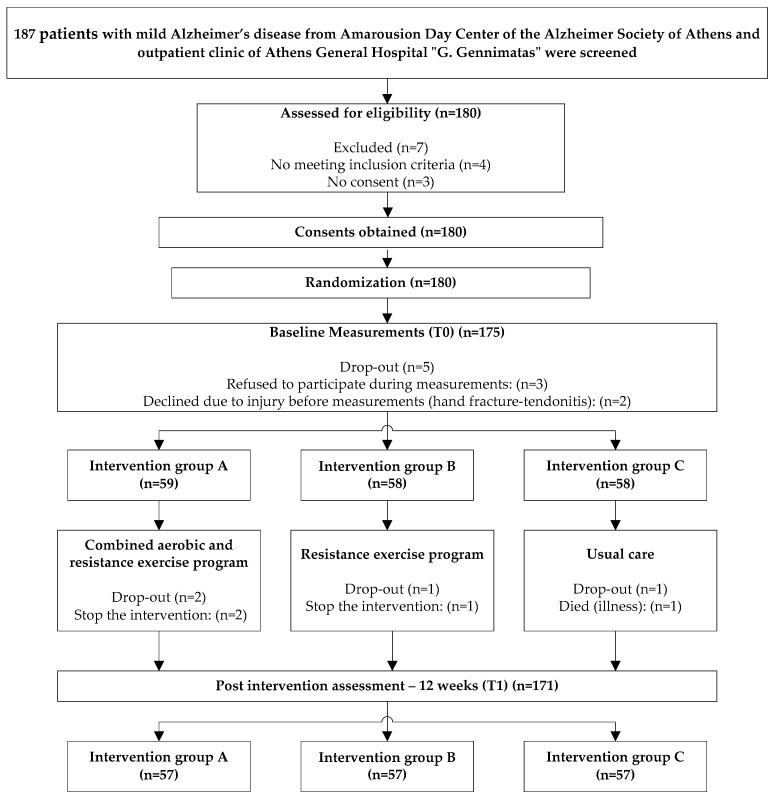
Flowchart of the study design and patient flow in accordance with Consolidated Standards of Reporting Trials (CONSORT).

The study was conducted in accordance with the Declaration of Helsinki and the protocol was approved by the Ethics Committee of the University of Western Attica, Athens, Greece. The protocol of the randomized controlled trial has been described in detail [35]. The sample was recruited from outpatients of the Amarousion Day Care Center of the Alzheimer Society of Athens and the Athens General Hospital “G. Gennimatas”. Study participants, after having been informed, signed a consent form to participate in the research. A total of 171 patients completed the intervention and final assessment. Inclusion criteria: age ≥ 65, mild AD, Mini-Mental State Examination (MMSE): 20–24/30, adequate hearing and vision, presence of the caregiver, medical consent to participate in exercise, absence of any other exercise program, no change of medication for at least 2 months and ability to consent. Exclusion criteria: neurological and/or other serious conditions, cancer, surgery during the previous year, and alcohol and/or drug abuse. Furthermore, the sample size was estimated using G*Power software (Version 3.1.9, Erdfelder, Faul, & Buchner, Dusseldorf, Germany) [36]. The sample size was determined using a priori power calculations (power of 0.80, alpha level of 0.05, 3 groups, 2 measurements) and the expected effect size (small to moderate) based on the primary outcome of cognition from comparable studies [37,38]. All the above are described in the flowchart (Figure 1).

### 2.1. Procedure

Randomization was performed by an independent investigator. Participants were allocated to one of three groups, using a random number generator, prior to baseline assessment. Furthermore, the allocation sequence was concealed from the relevant study investigators, as was the intervention. Participants were assessed before the start of the intervention and immediately after 12 weeks. Both participants and examiners were prohibited from providing information on the exercise program. Assessors were unaware of previous test results. The participants were divided into three intervention groups, group A (aerobic and resistance exercise, *n* = 57), group B (resistance exercise, *n* = 57), and group C (control group, *n* = 57).

The primary objective of the study is to determine the effects of exercise on cognition and organic activities of daily living in people with AD using assessment tools such as Addenbrooke’s Cognitive Examination-Revised (ACE-R), Trail Making Test A (TMT-A), Trail Making Test B (TMT-B), Digit Span Test Forward (DST-F) and Digit Span Test Backward (DST-B) and Instrumental Activities of Daily Living Scale (IADLs).

#### Tools

The tools used to assess the patients before and after the intervention were:

(a) The Addenbrooke’s Cognitive Examination-Revised (ACE-R) is a brief cognitive assessment tool that can be used alone in any cognitive assessment and takes approximately 20 min [39,40]. ACE-R is a very sensitive and specific test for the diagnosis of dementia [39,40]. It was designed to briefly examine a wide range of cognitive domains: attention/orienting, memory, fluency, language, and visuospatial [39,40]. The maximum ACE-R score is 100 (refers to the best cognitive function) [40,41].
(b) Trail Making Test A-Β is an easy and quick neuropsychological test that assesses cognitive abilities such as attention, processing speed, and executive functions [42]. The TMT consists of two parts, A and B, during which the examinee is instructed to quickly connect a set of 25 dots (in TMT-A all numbers and in TMT-B alternating numbers and letters) [42,43]. TMT-A assesses attention and processing speed [43] and TMT-B assesses executive functions [43,44,45].
(c) Digit Span Test (Forward and Backward) is one of the most common tests to assess attention and working memory (recent memory is assessed) [46,47]. The DST-F assesses attention [48] and DST-B assesses working memory [48]. The total kernel is the sum of the number of digits from the forward iteration and the number of digits from the reverse iteration [46,47].
(d) Instrumental Activities of Daily Living Scale (IADLs) is a suitable tool for assessing independent living skills in both healthy elderly and elderly persons with dementia [49,50,51,52,53]. It is easy to administer; the time is 10–15 min [52]. The scale measures eight domains of functioning: telephone use, shopping, food preparation, housekeeping, laundry, mode of transportation, responsibility for personal medication, and ability to manage finances [49,51,52]. The total score ranges from 0 to 8 for women and 0 to 5 for men [49,51,52].

### 2.2. Intervention

The intervention included three groups. The first intervention group (group A, *n* = 57) performed a combined program of aerobic and resistance exercise, the second (group B, *n* = 57) only resistance exercise, and the third (group C, *n* = 57) was the control group. The intervention program of groups A and B had different duration and frequencies for each type of exercise but with moderate intensity for both groups.

In the combined program intervention group A (aerobic and resistance exercise) the aerobic exercise was performed at home and included walking for a duration of 30 min, frequency of five days per week, and moderate intensity which is defined as 64–76% of the HRmax [22,26,54,55,56,57]. The resistance exercise concerned major muscle groups with limb weights at moderate intensity according to 50–69% of a maximum repetition (% 1-RM) [55,57]. The resistance exercise included 2 sets of 10 exercises of 12 repetitions [55,57]. The frequency pertained to three workouts per week (every 48 h), with a duration of 40–45 min per session, with a break of 1–3 min between sets [55,56,57,58,59,60,61]. One of the three days of resistance training was performed at home while the other two were performed at the participants’ recruitment center under the supervision of a physical therapist.

Group B performed only resistance exercise. The exercise program with resistance was the same for both intervention groups (group A and group B). Resistance exercises included most of the major muscle groups such as bicep arm curl, shoulder flexion, shoulder abduction, shoulder extension/hyperextension, triceps extension, hip flexion, knee extension, hip abduction, hip extension, and hamstring curls.

The participants in group C maintained their usual daily activity without participating in any exercise program.

Caregivers supervised the exercise sessions performed at home according to guidelines as well as monitored adherence to the exercise diary. Moreover, the sessions were monitored periodically by physical therapists. Initially, (during the first two weeks) home visits by physiotherapists were performed once a week, which was then reduced to one visit every four weeks. In addition, a telephone consultation was conducted once a week during the first four weeks and, subsequently, one call every four weeks until the end of the intervention. At the end of each month, the exercise diary form that had been provided was returned fully filled out.

### 2.3. Statistical Analysis

Despite the fact that we had a total of 171 subjects, 57 per group, all variables under consideration were tested for normality with the Kolmogorov–Smirnov test, and data were investigated for outliers. The frequency distributions for our measurements look plausible: we did not see any very low or high values. Associations between total changes of IADL score and measures of cognitive function (ACE-R and its subscales, TMT A and B, and Digit Span Test Forward and Backward) were assessed by Pearson’s correlation coefficient. To compare differences in characteristics between the three groups at baseline, one-way ANOVA F-tests were carried out and the chi-squared test (χ2) was applied to sets of categorical data. In addition, Bonferroni post hoc multiple pairwise comparison tests were performed in order to define between which groups were found statistically significant differences.

In order to determine group effects in each measurement, after the twelve-week-long intervention, Analyses of covariance (ANCOVA) were performed with cognitive and instrumental activities scores on the post-tests (T1) as dependent variables, pretest (T0) scores as covariates, and group (aerobic and resistance exercise, resistance exercise, and control) as between the subject factor. Furthermore, to specify significant group effects, ANCOVA Bonferroni corrected post hoc tests were done.

For each outcome measure in cognitive, mood, and physical function, mean Cohen’s d effect sizes were calculated by using the exercise group (aerobic and resistance or resistance) as the experimental and the control as the reference group. The following formula was used: d = [(post_exp._ − pre_exp._) − (post_ref._ − pre_ref._)]/√[([s^2^pre_exp_. (n_exp._) + s^2^pre_ref._ (n_ref._)]/[n_exp._ + n_ref._]) + ([s^2^post_exp._ (n_exp._) + s^2^post_ref._ (n_ref._)]/[n_exp._ + n_ref._ ])/2] [38].

Note: exp. = experimental group, ref. = reference group, post= mean score of post-test, pre = mean score of pretest, and s = standard deviation.

The effect size for each test shows a significant difference compared with Cohen’s guidelines (0.2 indicates a small effect, 0.5 indicates a moderate effect, and 0.8 indicates large effect sizes).

Results of the patients’ characteristics who participated in the research appear in mean and standard deviation (mean ± SD), while those of categorical data in frequencies *n* and percentages (%). *p*-values less than 0.05 were considered statistically significant. Statistical analyses were carried out using the software package SPSS 24.0 for Windows (SPSS Inc., Chicago, IL, USA).

## 3. Results

A total of 171 patients (45 men/126 women) with a mean age 77.22 ± 5.73 (65–91), BMI 27.20 ± 3.65 (12–36), and mean education years 14.08 ± 2.07 (11–18), diagnosed with mild dementia were analyzed. Analysis of Variance (ANOVA) for the three groups of patients revealed no significant differences in mean values for age, BMI, and education years (Table 1). No significant differences were found also between the three groups for the categorical variables, dementia, and depression medication, from the chi-square tests. Table 1 presents the patients’ characteristics for each group.

Table 2 presents the baseline measurements for each cognitive domain, as well as for instrumental activities data for each group. Analysis of variance (ANOVA) between groups of patients, at baseline, in cognitive measurements revealed significant differences for the total ACE-R score and its subscales: attention and orientation, memory, and visual spatial ability. No significant differences for total DST F-B and TMT A-B scores and the rest of the ACE-R subscales: verbal flow and language. Similarly, no significant differences were found for IADL.

Pearson correlation coefficients for the total changes show that cognitive function was significantly correlated with IADL. Specifically, IADL was positively and significantly correlated with ACE-R total (r = 0.539, *p* < 0.01) and its components attention and orientation (r = 0.478, *p* < 0.01), memory (r = 0.513, *p* < 0.01), verbal flow (r = 0.379, *p* < 0.01), language (r = 0.394, *p* < 0.01), and visual spatial ability (r = 0.426, *p* < 0.01). Similarly, IADL was found to have a significant moderate positive relation with DST total (r = 0.494, *p* < 0.01) and its components DST Forward (r = 0.455, *p* < 0.01) and DST Backward (r = 0.433, *p* < 0.01). Moreover, a significant negative correlation was found between IADL with TMT_A (r = −0.435, *p* < 0.05), and TMT_B (r = −0.205, *p* < 0.01).

Results from analyses of covariance for each measurement after the twelve-week-long intervention, with the baseline score as covariate, are shown in Table 3. A significant intervention effect was observed for all outcome measures (general cognitive function, and instrumental activities). ANCOVA Bonferroni corrected post hoc tests revealed that the aerobic and resistance group improved compared to the control group on all measurements scales. The resistance group also showed an improvement compared to the control. No significant effects were found between the aerobic and resistance group and the resistance group in any of the outcome measures. Table 4 presents the aforementioned results with *p*-values and 95% confidence intervals for the mean differences as well as the corresponding Cohen’s d.

## 4. Discussion

Our study sample consists of more women than men (Table 1). Indeed, the proportion of females with Alzheimer’s disease is higher than males [4].

To our knowledge, this study is the first to compare the effect of therapeutic exercise through a combined type of aerobic and resistance exercise and a single type of resistance exercise. Our findings demonstrate the existence of statistically significant differences in both types of therapeutic exercise in all subjects examined when compared to the control group.

Specifically, regarding global cognitive functioning (CF), the first group, compared to the control group, appeared to have a larger effect on the ACE-R total and subscales, such as verbal fluency, language, and visuospatial ability, than the second group (Cohen’s d 1.68 vs. 1.47, 0.85 vs. 0.73, 0.96 vs. 0.81, and 1.08 vs. 1.07 respectively). In contrast, the effect on the attention and orientation subscales in the resistance exercise group was larger (Cohen’s d 1.52 vs. 1.38), whilst, in terms of memory, both groups had the same score (0.75). In addition, more effect is observed in the first group than in the second group as regards attention (TMT-A, DST-F), processing speed (TMT-A), and working memory (DST-F). The second group seems to excel in executive functions (TMT-B) and instrumental activities of daily living (IADL scale).

Our study results could agree with Mcleod, et al. (2019), that resistance exercise training (RET) and aerobic exercise training (AET) do not lead to distinct health benefits; however, a contrary view is, in fact, pertaining to the larger amount of data currently available for AET as opposed to RET [62].

After the twelve weeks of intervention, the baseline values improved, which proves that a structured exercise program brings positive results and that continued exercise over time can delay the progression of AD.

The benefits of exercise in the geriatric population are well known. Yoon, et al. (2018), in a randomized controlled trial with a population of older adults with cognitive impairment (absence dementia) with resistance exercise intervention statistical analysis, showed that it significantly improved performance on tests of cognitive function (processing speed and executive function, *p* < 0, 05) [63].

Moderate physical activity (PA) and exercises have a positive impact on global cognitive function and working memory and attention, which is in agreement with our study [64]. Resistance exercise, along with aerobic exercise, should be an integral component of any exercise intervention aimed at improving health in any population [65]. Strength training as a therapeutic intervention for elderly persons with cognitive impairment and dementia is proposed not only for its beneficial effects on physical variables, such as increased muscle mass and strength or increased balance but also on cognitive function (memory and executive function) [59].

Exercise slows the decline and improves the cognitive function in patients with Alzheimer’s disease [26,57,59,66,67].

Li, et al. (2018) report in a systematic review that resistance training had positive effects on global cognition and executive function among older adults [68]. Aerobic exercise and resistance exercise were shown to be more effective in slowing cognitive function, global cognition, visual memory, verbal memory, and executive function in patients with dementia compared to the control group [38].

According to the systematic review by Guitar, et al. (2018), physical exercise can be effective in improving executive function in older adults living with dementia of the Alzheimer type; this statement is also consistent with our research results. Four of the six studies reviewed showed significant improvement (one intervention consisting of resistance training and three combined with aerobic, strength training, and flexibility or balance components) for the executive function of persons with AD [59,69]. Other studies report that resistance exercise is a powerful physical intervention strategy for inducing significant brain functional changes accompanied by improvements in executive functions [70].

Demurtas, et al. (2020) reports in a review that mixed physical activity/exercise was effective in improving global cognition in AD, which is supported by our research [71].

Vidoni, et al. (2019) shows that aerobic exercise maintains IADL independence in individuals in the earliest stages of AD [18]. IADL performance was not associated with sustained or improved executive function but with memory change [18]. Physical inactivity is associated with an increased incidence of dementia [72], and loss of ADL independence [18], and is one of the most important modifiable risk factors for dementia [32].

The results of the Liu, et al. (2020) study clearly indicate that both strength and aerobic training programs can bring about significant benefits for patients with dementia in both their activities of daily life (ADL) and cognitive function [37]. Furthermore, in the same study, the difference between aerobic and strength training was not significant; as such, our own study showed that the difference between the aerobic and resistance exercise interventions and the resistance exercise intervention was indeed not significant [37]. Finally, as was exhibited from our study as well, whether it is strength training or aerobic exercise, it is proven that both bring significant benefits to patients with dementia [37].

Physical exercise has equally been associated with a slower progression of functional limitations and, therefore, slower progression regarding the activity of daily living (ADL)/instrumental activity of daily living (IADL) disability [57]. Bossers, et al. (2016) reports that exercise can improve activities of daily living (ADL) in individuals with dementia and that a combined program of aerobic and strength training can be more beneficial than a program with one type of exercise (e.g., only aerobic exercise) [9]. Garuffi, et al. (2012) declare that resistance training is effective in improving basic and instrumental ADL performance in AD patients [8]. Patients in the resistance training group showed significantly improved performance in daily tasks requiring flexibility, lower extremity strength, balance, and flexibility and improving their autonomy and motor functionality, which are increasingly impaired with disease progression [8].

Our study results are compatible with the study of Klemmensen, et al. (2020) that a significant and positive relationship exists between cognitive function, (including processing speed and attention) and IADL in persons with mild AD [7].

Cognitive function is important for instrumental activities of daily living and interventions targeting cognitive function may also have a positive effect on IADL in individuals with mild AD [7].

The mechanisms involved in the cognitive benefits associated with exercise are not known [59,73], possibly due to increased cerebral blood volume and capillarization, levels of brain-derived neurotrophic factors and hippocampal volume, or a decrease in oxidative stress, inflammatory processes, cardiovascular conditions, or stress [59]. Moreover, resistance exercise requires the persons to perform complex cognitive tasks, such as comprehending the instructions, moving the limbs in the correct order, and mimicking [38]. In addition, strength exercises incorporate motor-coordination and balance tasks, leading to the activation of specific cerebellar–cortical connections, which can act as a stimulus for concurrent improvements in cognitive function and balance [38]. Machado, et al. (2017) mention that physical exercise of moderate intensity should be considered as a standard recommendation to reduce cognitive decline in patients with AD due to the improvement in neurodegenerative mechanisms and the increase in neuroplastic and neuroprotective neurotrophic factors [74].

Huang, et al. (2022), in their systematic review and network meta-analysis, report that executive function shows a significant effect with resistance exercise, which is also consistent with our study [75]. According to the same researchers, resistance exercise deserves greater importance as an adjunctive treatment for patients with cognitive dysfunction (e.g., dementia), given its significant benefits on cognitive function and knowledge-related outcomes such as ADL [75]. Multicomponent exercise such as aerobic and resistance training may be more beneficial than training in a single modality [76]. The findings support WHO recommendations to emphasize resistance training as a critical component of interventions for older adults [77,78].

Our findings highlight the effectiveness of resistance exercise in improving or slowing cognitive decline and IADL in patients with dementia, as well as its important role in combination with aerobic exercise. Both types of exercise (interventions) were effective in improving global cognitive function, executive function, and working memory, as well as instrumental daily activities in patients with dementia (AD), with mixed exercise being the most effective intervention.

Further investigation is needed and it may be recorded in future measurements whether or not these results are maintained over time.

## 5. Conclusions

Therapeutic exercise through aerobic exercise and resistance exercise can improve both cognitive functions and instrumental daily activities in patients with mild Alzheimer’s disease. Therefore, we recommend that older persons with dementia choose their preferred types of exercise to get the benefits of therapeutic exercise. Physiotherapists as health professionals are the only ones who are certified and have the necessary qualifications to train and support therapeutic exercise in neurological clinical populations [18]. More research is needed to obtain recommendations regarding the use and prescription of physical exercise as a therapeutic strategy in patients with Alzheimer’s disease.

## Figures and Tables

**Table 1:** Personal characteristics for each group at baseline. Age, BMI, and education years are presented as the mean ± sd and all categorical data presented as the frequency and (%) values.

		Aerobic and Resistance Group (*n* = 57)		Resistance Group (*n* = 57)		Control Group (*n* = 57)		F/Chi2 Test Value, *p*-Value
Age		76.82	±5.73	76.07	±5.7	78.75	±7.06	F = 2.85, *p* = 0.061
BMI		27.39	±3.54	27.06	±3.65	27.15	±3.81	F = 0.122, *p* = 0.885
Education Years		14.07	±2.00	14.54	±2.08	13.61	±2.06	F = 2.93, *p* = 0.056
Gender	man	21	(36.8)	17	(29.8)	7	(12.3)	Chi2 = 9.41, *p* = 0.009
	woman	36	(63.2)	40	(70.2)	50	(87.7)	
Dementia Medication	no	33	(57.9)	31	(54.4)	26	(45.6)	Chi2 = 1.83, *p* = 0.401
	yes	24	(42.1)	26	(45.6)	31	(54.4)	
Depression Medication	no	47	(82.5)	40	(70.2)	45	(78.9)	Chi2 = 2.59, *p* = 0.274
	yes	10	(17.5)	17	(29.8)	12	(21.1)	

Differences between groups were tested with one-way analysis of variance (ANOVA) and for categorical data with Pearson’s Chi2 test.

**Table 2:** Measurements characteristics at baseline for each group.

	Aerobic and Resistance Group (*n* = 57)		Resistance Group (*n* = 57)		Control Group (*n* = 57)		F Value, *p*-Value
ACE-R total	74.04 ^a,c^	±7.42	70.51 ^c^	±8.83	70.53 ^a^	±6.80	F = 3.93, *p* = 0.021
ACE-R Attention and Orientation	14.53 ^a,c^	±2.30	13.46 ^c^	±1.77	13.25 ^a^	±1.96	F = 6.56, *p* = 0.002
ACE-R Memory	17.18	±4.28	15.93 ^b^	±4.27	18.19 ^b^	±3.33	F = 4.61, *p* = 0.011
ACE-R Verbal flow	8.19	±2.1	8.68	±1.97	7.75	±2.12	F = 2.89, *p* = 0.059
ACE-R Language	20.84	±2.84	20.25	±2.91	20.00	±2.82	F = 1.43, *p* = 0.242
ACE-R Visual Spatial Ability	13.3 ^a,c^	±1.56	12.19 ^c,b^	±2.06	11.33 ^a,b^	±1.83	F = 16.61, *p* < 0.001
Trial Making Test A	132.5 ^a,c^	±61.04	165.56 ^c^	±71.1	175.30 ^a^	±59.7	F = 7.01, *p* = 0.001
Trial Making Test B	235.7 ^a,c^	±66.46	264.07 ^c^	±55.1	280.35 ^a^	±39.3	F = 9.70, *p* < 0.001
DST Forward	5.16	±0.87	5.21	±1.18	4.98	±0.92	F = 0.82, *p* = 0.441
DST Backward	3.00	±1.24	3.07 ^b^	±1.08	2.56 ^b^	±0.98	F = 2.85, *p* = 0.031
DST total	8.16	±1.6	8.28	±2.01	7.54	±1.65	F = 2.85, *p* = 0.061
IADL	5.93	±1.76	5.61	±1.85	5.56	±1.67	F = 0.73, *p* = 0.484

Differences between groups were tested with one-way analysis of variance (ANOVA). Post hoc Bonferoni multiple comparison results. ^a^. significant difference between the aerobic & resistance and control group. ^b^. significant difference between the resistance and Control group. ^c^. significant difference between the aerobic & resistance and resistance group.

**Table 3:** Measurement characteristics for each group after the intervention.

	Aerobic and Resistance Group (*n* = 57)		Resistance Group (*n* = 57)		Control Group (*n* = 57)		F Value, *p*-Value
ACE-R total	79.25 ^a^	±6.46	75.7 ^b^	±8.61	64.28 ^a,b^	±6.51	F = 160.3, *p* < 0.001
ACE-R Attention and Orientation	15.60 ^a^	±1.70	14.67 ^b^	±1.91	11.65 ^a,b^	±1.74	F = 152.3, *p* < 0.001
ACE-R Memory	18.60 ^a^	±4.16	17.33 ^b^	±4.12	16.75 ^a,b^	±3.38	F = 50.1, *p* < 0.001
ACE-R Verbal flow	9.35 ^a^	±1.87	9.63 ^b^	±2.19	7.19 ^a,b^	±2.01	F = 50.67, *p* < 0.001
ACE-R Language	21.82 ^a^	±2.22	20.98 ^b^	±2.95	18.47 ^a,b^	±2.49	F = 70.1, *p* < 0.001
ACE-R Visual Spatial ability	13.88 ^a^	±1.19	13.07 ^b^	±1.90	10.21 ^a,b^	±1.63	F = 92.16, *p* < 0.001
Trial Making Test A	110.1 ^a^	±59.48	147.2 ^b^	±70.8	219.6 ^a,b^	±59.73	F = 83.21, *p* < 0.001
Trial Making Test B	218.3 ^a^	±69.32	248.5 ^b^	±58.7	291.8 ^a,b^	±27.9	F = 21.02, *p* < 0.001
DST Forward	5.86 ^a^	±0.93	6.02 ^b^	±0.92	4.25 ^a,b^	±1.04	F = 90.9, *p* < 0.001
DST Backward	3.77 ^a^	±1.09	3.53 ^b^	±1.10	1.33 ^a,b^	±1.06	F = 112.1, *p* < 0.001
DST total	9.63 ^a^	±1.75	9.54 ^b^	±1.80	5.58 ^a,b^	±1.87	F = 141.3, *p* < 0.001
IADL	6.04 ^a^	±1.71	5.86 ^b^	±1.73	4.14 ^a,b^	±1.82	F = 87.71, *p* < 0.001

Differences between groups were tested with a one-way analysis of covariance (ANCOVA). Post hoc Bonferroni multiple comparison results. ^a^. significant difference between the aerobic and resistance and control group. ^b^. significant difference between the resistance and control group.

**Table 4:** Results of ANCOVA Bonferroni multiple comparisons tests with Cohen’s d (standardized effect size).

	Mean Difference	Std. Error	*p*-Value ^b^	95% CI for Difference ^b^ Low B.Up B.		Cohen’s d
ACE-R total						
Between A and B group	0.806	0.772	0.894	−1.06	2.67	0.01
Between A and C group	12.223 *	0.772	0.000	10.36	14.09	1.68
Between B and C group	11.417 *	0.759	0.000	9.58	13.25	1.47
ACE-R Attention and Orientation						
Between A and B group	0.154	0.196	1.000	−0.32	0.63	−0.07
Between A and C group	3.018 *	0.198	0.000	2.54	3.50	1.38
Between B and C group	2.865 *	0.192	0.000	2.40	3.33	1.52
ACE-R Memory						
Between A and B group	0.152	0.306	1.000	−0.59	0.89	0.00
Between A and C group	2.750 *	0.305	0.000	2.01	3.49	0.75
Between B and C group	2.597 *	0.312	0.000	1.84	3.35	0.75
ACE-R Verbal flow						
Between A and B group	0.133	0.197	1.000	−0.34	0.61	0.10
Between A and C group	1.789 *	0.197	0.000	1.31	2.26	0.85
Between B and C group	1.656 *	0.199	0.000	1.17	2.14	0.73
ACE-R Language						
Between A and B group	0.356	0.244	0.439	−0.23	0.95	0.09
Between A and C group	2.665 *	0.245	0.000	2.07	3.26	0.96
Between B and C group	2.309 *	0.243	0.000	1.72	2.90	0.81
ACE-R Visual Spatial ability						
Between A and B group	0.038	0.188	1.000	−0.42	0.49	−0.18
Between A and C group	2.299 *	0.200	0.000	1.82	2.78	1.08
Between B and C group	2.261 *	0.186	0.000	1.81	2.71	1.07
Trial Making Test A						
Between A and B group	−6.852	6.022	0.770	−21.42	7.71	−0.06
Between A and C group	−70.397 *	6.110	0.000	−85.17	−55.62	−1.11
Between B and C group	−63.544 *	5.902	0.000	−77.82	−49.27	−0.96
Trial Making Test B						
Between A and B group	−6.534	5.858	0.799	−20.70	7.63	−0.03
Between A and C group	−36.310 *	6.043	0.000	−50.92	−21.70	−0.54
Between B and C group	−29.776 *	5.772	0.000	−43.73	−15.82	−0.58
DST Forward						
Between A and B group	−0.123	0.133	1.000	−0.45	0.20	−0.11
Between A and C group	1.499 *	0.134	0.000	1.18	1.82	1.52
Between B and C group	1.622 *	0.134	0.000	1.30	1.95	1.51
DST Backward						
Between A and B group	0.291	0.153	0.179	−0.08	0.66	0.27
Between A and C group	2.157 *	0.155	0.000	1.78	2.53	1.82
Between B and C group	1.866 *	0.156	0.000	1.49	2.24	1.60
DST_Total						
Between A and B group	0.177	0.238	1.000	−0.40	0.75	0.12
Between A and C group	3.607 *	0.241	0.000	3.03	4.19	1.99
Between B and C group	3.430 *	0.242	0.000	2.85	4.01	1.75
IADL						
Between A and B group	−0.109	0.141	1.000	−0.45	0.23	−0.08
Between A and C group	1.563 *	0.142	0.000	1.22	1.91	0.88
Between B and C group	1.672 *	0.141	0.000	1.33	2.01	0.94

Based on estimated marginal means. *. The mean difference is significant at the 0.05 level. ^b^. Adjustment for multiple comparisons: Bonferroni.

## Data Availability

The data are not publicly available due to privacy restrictions.
